# Vitamin D deficiency mediates the relationship between dietary patterns and depression: a case–control study

**DOI:** 10.1186/s12991-020-00288-1

**Published:** 2020-06-03

**Authors:** Gity Sotoudeh, Firoozeh Raisi, Maryam Amini, Reza Majdzadeh, Mahdieh Hosseinzadeh, Fatemeh Khorram Rouz, Maryam Khosravi

**Affiliations:** 1 Department of Community Nutrition, School of Nutritional Sciences and Dietetics, Tehran University of Medical Sciences, Tehran, Iran; 2 Department of Psychiatry, Roozbeh Hospital and Psychiatry and Psychology Research Centre, Tehran University of Medical Sciences, Tehran, Iran; 3 Department of Nutrition Research, Faculty of Nutrition Sciences and Food Technology, National Nutrition and Food Technology Research Institute, Shahid Beheshti University of Medical Sciences, Tehran, Iran; 4 Knowledge Utilization Research Center and Community Based Participatory Research Center, Tehran University of Medical Sciences, Tehran, Iran; 5 Department of Nutrition, Shahid Sadoughi University of Medical Sciences, Yazd, Iran; 6 Student Research Committee, Department of Nutrition, School of Medicine, Mashhad University of Medical Sciences, Mashhad, Iran; 7 Department of Nutrition, School of Medicine, Mashhad University of Medical Sciences, Vakilabad Blv. Azadi Squre, Mashhad, Iran; 8 Department of Public Health, North Khorasan University of Medical Sciences, Bojnurd, Iran

**Keywords:** Depression, Dietary pattern, Vitamin D, Zinc, Magnesium, Total antioxidant capacity, Mediatory analysis

## Abstract

**Background:**

Depression is a major contributor to disability-adjusted life years (DALY) lost in the world. Dietary patterns are widely used to investigate diet–disease relations. In the current study, the relationship between dietary patterns and depression was investigated. Besides, the role of serum vitamin D, zinc, magnesium, and total antioxidant capacity as potential mediatory variables was studied.

**Methods:**

It was an individually matched case–control study in which 330 depressed and healthy subjects were recruited for the extraction of dietary patterns; psychiatrists diagnosed major depressive disorder, using the criteria of the Diagnostic and Statistical Manual of Mental Disorders. Serum vitamin D and aforementioned biomarkers were measured for a number of randomly selected depressed and healthy individuals. We conducted mediatory analysis by regression models.

**Results:**

Healthy and unhealthy dietary patterns were associated with the lower and higher odds of depression (OR 0.39, CI 0.17–0.92 and OR 2.6, CI 1.04–6.08), respectively. A significant relationship between serum vitamin D with depression after adjusting for potential confounders was observed as well (OR 0.93, CI 0.87–0.99). According to the mediatory analysis the unhealthy dietary patterns were related to depression via altering the serum vitamin D concentration.

**Conclusion:**

This study showed that vitamin D deficiency mediates the relationship between unhealthy dietary patterns and depression. However, to get a clearer result further prospective studies are required.

## Background

Depression is a common heath problem all over the world which currently affects 264 million people [1]. It is a leading cause of disability worldwide and is a major contributor to DALY lost in the world [2]. Depression is highly prevalent in developed and developing countries. In the USA, it has been estimated that 6.3% of adult population are depressed [3]. Depression ranks the forth contributing factor for burden of disease and is anticipated to rank the second until 2020. In Iran, depression constitutes 35% to 45% of mental illnesses and about 8% to 20% of the population suffer from it [4]. There are some medications for treatment of depression such as drugs that increase serotonin concentration as tricyclic antidepressants (TCAs), monoamine oxidase inhibitors (MAOIs) and serotonin-specific reuptake inhibitors (SSRIs) which are reported to have either side effects or not to be effective enough [5].

Diet has long shown to contribute to the treatment of depression. Prior studies on diet–depression associations have mostly focused on nutrients [6–8], foods [9] and food groups [10] rather than dietary patterns. Due to the complex interactions between nutrients and foods, nutritional epidemiologists have suggested to deploy dietary pattern approach in investigating diet–disease relations [11]. This approach can provide a more comprehensive and new insight toward the diet–mental health relations [12]. Some studies have assessed the association between dominant dietary patterns and risk of depression [13–15] which most of them have been driven from food questioners and not biochemical assessment. Some potential biomarkers like 25(OH) D, total antioxidant capacity (TAC), zinc (Zn), and magnesium (Mg) in serum are reported to be significantly related to depression. For example, low levels of serum 25(OH) D were associated with depressive symptoms [16], and in a large cohort study it was concluded that hypovitaminosis D may increase vulnerability for depression [17]. According to another study vitamin D-deficient people had higher risk for depression [18] which was confirmed by a meta-analysis [19]. It is documented that vitamin D has some receptors in the hypothalamus [20], and plays an important role in brain development [21]. Furthermore, case–control studies have shown TAC concentration is lower in depressed people compared with their healthy counterparts, as well as a negative dose–response relation observed between depression severity and TAC [22]. According to several studies a negative relationship was seen between zinc and depression [23, 24], and magnesium showed to have a protective effect in treatment of depression via glutamate system [25], neurotransmitter metabolism [23] and psychomotor function [26].

So far it is not established if biochemical ingredients mediate the relation between dietary patterns and depression. So, in the current study we aimed to investigate the mediatory role of all above-mentioned biomarkers in the relation of dietary patterns and depression.

## Methods

We acknowledge that the paper has been compliance with STROBE checklist.

### Participants and study design

A total of 110 depressed patients and 220 healthy individuals participated in this individually matched case–control observational study. Serum vitamin D, zinc, magnesium, and total antioxidant capacity were considered as potential mediatory variables, when evaluating the relationship between dietary patterns and depression. The patients were selected from two psychiatric clinics in Tehran. For recruitment of controls, we reached each patient’s residential area, and invited eligible people to participate in the study.

According to the criteria of the Diagnostic and Statistical Manual of Mental Disorders-IV, the patients’ diseases were diagnosed by the psychiatrist as major depressive disorder [27]. No one in the case and control groups did not have history of depression in the past year. Individual matching between two groups was done, based on sex, age, and residential area. Each people with depression was matched with two people as control within 10-year age categories.

For evaluating the relationship between biochemical markers and depression, the sample size was calculated for each quantitative biochemical marker separately and the highest obtained sample size was 43 matched cases and controls (86 depressed and healthy people out of total cases and controls). They were randomly selected for biochemical analysis including serum levels of 25(OH) D, TAC, Zn, and Mg. It is notice worthy that in this study biases were minimized by matching, considering precise criteria for inclusion and exclusion, correct selection of the case and control participants and using new cases.

#### Inclusion criteria

People aged 18–65 years, residing in Tehran, having major depressive disorder with a maximum period of 3-month intervals from onset of five symptoms of depression to the beginning of the study were included in the case group. For inclusion of the control group, the criterion was the absence of major depressive disorder, based on Beck Depression Inventory questionnaire (BDI-II), standardized in Iran [28].

#### Exclusion criteria

People who suffered from cognitive impairment or other psychotic illnesses diagnosed by a psychiatrist; those who had severe depression or lacked ability to cooperate and answer the questions; took any anti-depression drugs or treatments; suffered from hormonal disorders like Addison’s, Cushing’s disease; had hyperthyroidism, hypothyroidism, and hyperparathyroidism; suffered from chronic diseases like cancer, heart disease, diabetes, stroke, fibromyalgia, kidney or liver failure, multiple sclerosis and Parkinson disease; had history of trauma, cuts, fractures, bleeding, burns, accidents and other similar events in the past 3 months that resulted in unconsciousness and hospitalization; suffered from chronic and infectious diseases like HIV, mononucleosis, tuberculosis, viral hepatitis and pneumonia in the past 2 weeks; people who were addicted to alcohol and/or drug at the time of the study or in the past 3 months; had BMI ≥ 40 kg/m^2^, pregnancy and lactation at the time of the study or in the past year, any type of special diet in the past 2 months, any type of special diet for more than 2 months in the past year; took vitamin D more than once in the last 6 months; took Zn and Mg within at least 2 previous months; and people who took other nutritional supplements continuously, by injection or orally in the past month.

After describing the aim of the study, the written informed consent form was signed by all the participants. The study protocol was approved by the Ethics Committee of Tehran University of Medical Sciences. The ethics code was 19374-161-03-91.

### Assessment of covariates

A demographic questionnaire was employed to collect general information and some confounders. Anthropometric measurements were obtained from all subjects with a precision of 100 g for weight and 0.5 cm for height. Dietary intakes of the subjects in the last 12 months were assessed using a valid and reliable semi-quantitative food frequency questionnaire (FFQ) [29]. Physical activity was measured by a valid questionnaire in Iran [30]. The questionnaire consisted of nine levels of activity from rest and sleep (MET = 0.9) to vigorous activity (MET ≥ 6), based on the metabolic equivalent task hours per day (MET-h/day). An M.Sc. holder in nutrition collected data of physical activity. Depression was diagnosed based on the fourth edition of DSM criteria by a psychotherapist. For quantitative measurement of anxiety as a confounder, the Iranian standardized Beck Anxiety Inventory or BAI-II [31] was utilized. We used standardized Beck Depression Inventory questionnaire or BDI-II [28] for screening controls.

### Assessment of serum biomarkers

Blood samples were collected before patients took any antidepressant drugs. To measure biomarkers, 5 ml blood samples were collected from the subjects who fasted for 12 h, between 7 and 10 AM and transferred into tubes with no anticoagulant. After centrifuging for 20 min at 1500*g* in room temperature, the serum was separated and stored at − 70 °C. Serum 25(OH) D was assay by Enzyme immunoassay (EIA) method (IDS, UK). We measured serum total antioxidant capacity (TAC) with 3-ethylbenzothiazoline-6-sulfonic acid as a peroxidase substrate suitable for using in ELISA procedures. Serum Mg and serum Zn were measured with colorimetric assay (0.05–5 mg/dl pars azemun) and chemistry methods (Selecta E, Vitalab, Netherland in µg/dl), respectively.

### Statistical analysis

“Kolmogorov–Simonov test was applied to analyze the normality of covariates, followed by *t* test or Mann–Whitney test to compare variables in two groups. To compare qualitative variables Chi square was used. The exploratory factor analysis/principal component analysis was applied to determine the dietary patterns. According to the nutrient profiles and culinary recipes, food items of the FFQ were classified into 26 food groups. Food groups with factor loadings ≥ 0.3 were considered as important contributors to a dietary pattern. The factors were orthogonally converted using varimax rotation to improve interpretability. To identify whether a factor should be retained, the study factors were naturally interpreted in conjunction with eigenvalues that was equal to 1.5 and the scree plot was determined. The factor score for each person was calculated by summing the intakes of food groups weighted by his/her factor loading. The derived factors (two dietary patterns) were labeled based on our interpretation of the data and of the earlier literature. To identify the association of dietary patterns with other dependent variables, the calculated scores for each individual in each pattern were used as independent variables.

Finally, two dietary patterns, healthy (high in fruits, cruciferous, yellow, green leafy and other vegetables, low-fat dairies, whole grains, nuts, and olives) and unhealthy (high in refined grains and breads, high-fat dairy, solid oils, liquid oils and mayonnaise, pickles, snacks, soft drinks, industrial fruits and juice, red meats, poultry, processed meats, and sweets), were defined [32, 33].

Then, dietary patterns were used to evaluate the association of depression with dietary patterns and to adjust the confounders in multiple logistic regression. Multiple logistic regression models were used to assess the mediatory role of blood biomarkers related to depression. The following criteria were used to seek mediatory role of a variable [34]:

1. Significance of the relationship between dietary pattern(s) and depression;
2. No longer significance of the relationship between dietary pattern and depression after adding the mediatory variable to the model. In other words, after adding the mediatory variable(s) into the model, the relationship between dietary pattern(s) and depression had to transfer into the relationship between the mediatory variable and depression. Therefore, a third model had to be designed for ensuring the significant relationship between the mediatory variable(s) and depression.
3. Significance of the relationship between the mediatory variable and depression.

The mediatory analysis was performed after adjusting some confounding variables such as job, education, marital status, children number, energy intake, and so on. The mentioned covariates were related to both dietary patterns (as the independent variables) and depression (as the dependent variable) which indicated they were confounders. In this way, the mediatory variables were in the causal path of dietary pattern and depression [35].

All statistical analyses were carried out using SPSS (version 20; Chicago, IL).

## Results

According to Table 1 for some important variables including weight, height, age, energy intake, smoking, and hookah, there was not a significant difference between case and control group indicating the matching had been done correctly.

**Table 1:** Baseline characteristics of study population in case and control groups

Variables	Depressed patients	Control subjects	*p* value*
Height (cm)	162.81 ± 8.25	163.36 ± 8.67	0.59
Weight (kg)	69.76 ± 13.94	70.17 ± 14.24	0.8
Age	35.85 ± 10.86	35.69 ± 10.75	0.89
Energy (kcal)	2610 (2122–3293)	2477 (1917–3096)	0.06
Cigarette use			
Yes	11 (10)	12 (5.4)	0.11
No	98 (89.1)	209 (94.6)	
Hookah use			
Yes	6 (5.5)	17 (7.7)	0.46
No	102 (92.7)	203 (91.9)	
Education			
≤ Diploma	75 (68.2)	132 (59.7)	0.05
> Diploma	31 (28.2)	89 (40.3)	
Family number			
≤ 2	18 (16.4)	28 (12.7)	0.6
3–4	68 (61.8)	138 (62.4)	
≥ 5	24 (21.8)	55 (24.9)	
BMI			
≤ 18.5	3 (2.7)	22 (10)	0.03
18.5–24.9	48 (43.6)	76 (34.4)	
≥ 25	56 (50.9)	116 (52.5)	
Physical activity			
Mild	67 (60.9)	97 (43.9)	0.007
Moderate and severe	43 (39.1)	118 (53.4)	
Job status			
Housekeeper	66 (60)	99 (44.8)	0.01
Employee and student	21 (19.1)	80 (36.2)	
Free job	17 (15.5)	34 (15.4)	
Retired	6 (5.5)	8 (3.6)	
Life event			
Children event	19 (17.3)	33 (14.9)	< 0.001
Life event	28 (25.5)	30 (13.6)	
Both	20 (18.2)	17 (7.7)	
Children number			
≤ 2	83 (75.5)	192 (86.9)	0.02
3–4	23 (20.9)	27 (12.2)	
≥ 5	4 (3.6)	2 (0.9)	

Data were presented as frequencies and percentages for categorical variables and mean ± SD, median (Q1–Q3) for normally and non-normally distributed variables, respectively

*p values calculated by Chi square for categorical values and Independent samples *t* test or Mann–Whitney test for continuous values

Based on our published results [33], the healthy dietary pattern significantly was related to the lower odds ratio of depression (OR 0.39, CI 0.17–0.92), and the unhealthy dietary pattern significantly was related to the higher odds ratio of depression (OR 2.6, CI 1.04–6.08).

We observed a significant relationship between serum vitamin D (OR 0.93, CI 0.87–0.99) and TAC (OR 2.08, CI 1.17–3.72) with depression after adjustment for some potential confounders. However, there was no significant association between serum zinc and magnesium, and depression (Table 2).

**Table 2:** Compare of some serum biochemical factors in case and control groups

	Case (Mean ± S.E.)	Control (Mean ± S.E.)	OR (95% CI)
Vitamin D (nm/l)^d^	10.8 ± 1.1	15 ± 2	0.93 (*0.87–0.99*)
TAC^a^ (mmol/l)^d^	1.06 ± 0.025	0.99 ± 0.02	2.08 (*1.17–3.72*)
Zn^b^ (mg/dl)^e^	147.8 ± 4.2	150.3 ± 4.1	0.99 (0.97–1.01)
Mg^c^ (mg/dl)^e^	2.2 ± 0.05	2.21 ± 0.2	0.28 (0.03–2.88)

*p* < 0.05 values are in italic

^a^Total antioxidant capacity

^b^Zinc

^c^Magnesium

^d^Multiple logistic regression after adjusting for job, education, marital status, children number, smoking and hookah, depression history, unemployment history in past 5 year, tragic events in past 6 months, energy expenditure, and physical activity

^e^Multiple logistic regression after adjusting age, sex, non-depression drugs, smoking and hookah, history of depression, body mass index, energy expenditure, and physical activity

In addition, in mediatory analysis unhealthy dietary pattern was inversely related to depression via changing the serum level of vitamin D after adjusting for job, education, marital status, children number, smoking and hookah, depression history, unemployment history in past 5 year, tragic events in past 6 months, energy intake, and physical activity (Table 3).

**Table 3:** Logistic regression model for mediation analysis in the pathway of the relation of dietary patterns with depression

Model	1 *p* value	2 *p* value	3 *p* value	OR (95% CI)
Healthy dietary pattern				
Vitamin D (nm/l)	*0.028*	0.16	*0.07*	0.9 (0.86–1.006)
TAC (mmol/l)	*0.028*	0.5	0.2	Invalid model*
Unhealthy dietary pattern				
Vitamin D (nm/l)	*0.02*	0.3	*0.025*	0.89 (0.82–0.98)
TAC (mmol/l)	0.2	0.3	0.1	Invalid model*

*p* ≤ 0.05 values are in italic

Model 1: Logistic regression model for studying of relationship between depression and dietary patterns

Model 2: Logistic regression for studying of relationship between healthy dietary pattern and depression with mediation variables

Model 3: Logistic regression for studying of relationship between mediation variables and depression

All of three models adjusted for job, education, marital status, children number, unemployment history in past 5 year, tragic events in past 6 months r, smoking and hookah, depression history, energy expenditure, and physical activity

* Confidence intervals and *p* values for Hosmer and Lemeshow test that is the criterion of the goodness-of-fit for Logistic regression were nonacceptable

Model 1 in Table 3 illustrates the significant relation between dietary patterns and depression. For hypothesis testing of mediatory role, vitamin D was added in regression in model 2 (the same Table). By adding this variable, the significant relation between both dietary patterns and depression eliminated. In other words, the relationship between dietary patterns and depression moved to the relationship between the mediatory variable and depression. Therefore, it can be concluded that vitamin D is an intermediate variable. For confirmation of the idea, we examined the relation of vitamin D with depression (Table 3—model 3). This mediatory role could be established by the significant results of the latest model. It was concluded that only unhealthy dietary pattern is related to depression via the intermediary role of vitamin D. In other words, people who had an unhealthy diet, if their vitamin D was increased by one unit, their odds of depression would be reduced by 11%.

Complete mediation in which other exposures no longer affects outcome after intermediary variable was controlled. Based on the results, vitamin D was a complete mediator because after serum vitamin D had been entered the relationship between unhealthy dietary pattern and depression disappeared [36].

We repeated testing the model for TAC (Table 3) because TAC was significantly related to depression (Table 2). However, the goodness of fit for the logistic regression model (based on confidence intervals and *p* value for Hosmer and Lemeshow test), was not valid (Table 3). There was no significant association between depression and serum zinc and magnesium. Hence, we did not do mediatory analysis for them.

## Discussion

In our study, there was a significant relationship between depression and serum vitamin D as well as between the unhealthy dietary pattern and depression after adjustment for some potential confounders. There was also a mediatory role for vitamin D in the pathway between unhealthy dietary pattern and depression. Therefore, we concluded that if people on an unhealthy diet try to raise their serum vitamin D levels by consuming more vitamin D, the chance of depression will be reduced among them. To the best of our knowledge, it is the first study that evaluates mediatory role of serum vitamin D in the associations between dietary pattern and depression.

According to our findings hypovitaminosis D resulted in higher odds of depression. In accordance with the present study, Eyles et al. [37] showed that in rats whose mothers were vitamin D deficient their brain in terms of gross morphology, cellular proliferation, and growth factor signaling as well as expression of nerve growth factor was not developed properly. In contrast with the mentioned study, in a cross-sectional study conducted in middle-aged and elderly Chinese, depressive symptoms were not associated with 25(OH) D concentrations [38].

Several mechanisms have been proposed to explain the association between vitamin D and depression. Effects of active form of vitamin D (1, 25 dihydroxycholecalciferol) in brain tissue have been established by the detection of vitamin D receptors (VDR) in different parts of the brain [10, 39] such as amygdale as the center of the limbic system that affects behavior and emotions [40]. Vitamin D has also several neuroprotective functions, for example calcitriol regulates concentrations of calcium in neurons that could decrease toxicity resulted from excess calcium [13, 15, 18]. However, more studies are needed to examine the long-term effect of vitamin D depletion on the brain.

We could not establish the mediatory roles of TAC in the pathway of the relationship between the dietary pattern and depression because the goodness-of-fit criterion for the Logistic regression models was not acceptable (logical confidence intervals and *p* values for the Hosmer–Lemeshow test). Similar to the current study, Gonoodi et al. [41] did not find any significant relation between serum Zn levels and depression score in 408 adolescent girls aged 12–18 years. There were other studies which reported serum Zn concentrations did not differ between depressed patients and healthy group that support our results [42–44]. A randomized clinical trial demonstrated the efficacy of zinc supplementation in treatment of depression [45]. Moreover, one meta-analyses confirmed an inverse association between serum Zn concentration and depression [46]. All depressed patients in our study were new cases and it is the probable reason we could not observe any relationship in this regard. In other words, there was not enough time for reduction of zinc in the newly recognized patients. Another possible cause can be justified by the fact that the populations in different studies were not the same.

Results concerning evaluating serum magnesium concentrations in depressive disorders were inconsistent. Some authors found higher levels of serum Mg in depressed patient compared to healthy group [47, 48] which are in contrast to our finding. On the other hand, several studies reported an inverse association between serum Mg levels and depression [49–52]. However, it seems serum Mg levels may not be a proper indicator of depressive disorders [52].

### Strengths and limitations

In the current study we recruited new cases of depression. In addition, we conducted mediatory analysis, considered all inclusion and exclusion criteria precisely and minimized selection bias in the control group by going to the residential area of each patient.

The most important limitation of our study goes back to the nature of case–control studies in which the chance of recall bias is high, as well as the temporal relationship between depression and dietary patterns cannot be realized in such studies. Another limitation was related to financial restrictions which forced us not to do biochemical measurements for all the participants.

### Recommendations

Some oxidative stress biomarkers such as albumin, HDL cholesterol, and uric acid are likely to be associated with depression. Therefore, we highly recommend future studies which evaluate the mediatory role of the mentioned biomarkers in the relationship between dietary pattern and depression.

## Conclusion

This study showed that Vitamin D deficiency mediates the relationship between unhealthy dietary patterns and depression. However, to confirm the finding further prospective studies are suggested.

## Data Availability

The datasets produced and analyzed during the current study are not publicly available, but they are available from the corresponding author on reasonable request.
